# Vulnerability of SARS-CoV-2 and PR8 H1N1 virus to cold atmospheric plasma activated media

**DOI:** 10.1038/s41598-021-04360-y

**Published:** 2022-01-07

**Authors:** Osvaldo Daniel Cortázar, Ana Megía-Macías, Sandra Moreno, Alejandro Brun, Eduardo Gómez-Casado

**Affiliations:** 1 University of Castilla-La Mancha, Institute of Energy Research (INEI), C/Moledores s/n., 13071 Ciudad Real, Spain; 2 Mechanical Engineering Department, ICAI, Comillas Pontifical University, Alberto Aguilera 25, 28015 Madrid, Spain; 3 Institute for Research in Technology, ICAI, Comillas Pontifical University, Santa Cruz de Marcenado, 26, 28015 Madrid, Spain; 4 Animal Health Research Center (CISA, INIA-CSIC), National Research Institute of Agricultural and Food Technology (CSIC-INIA), Crta. de Valdeolmos-El Casar s/n - 28130, Madrid, Spain; 5 Department of Biotechnology, National Research Institute of Agricultural and Food Technology (INIA-CSIC), Crta. de la Coruña, km 7.5, 28040 Madrid, Spain

**Keywords:** Viral infection, Biological physics

## Abstract

Cold Atmospheric Plasma (CAP) and Plasma Activated Media (PAM) are effective against bacteria, fungi, cancer cells, and viruses because they can deliver Reactive Oxygen and Nitrogen Species (RONS) on a living tissue with negligible damage on health cells. The antiviral activity of CAP against SARS-CoV-2 is being investigated, however, the same but of PAM has not been explored despite its potential. In the present study, the capability of Plasma Activated Media (PAM) to inactivate SARS-CoV-2 and PR8 H1N1 influenza virus with negligible damage on healthy cells is demonstrated. PAM acted by both virus detaching and diminished replication. Furthermore, the treatment of A549 lung cells at different times with buffered PAM did not induce interleukin 8 expression, showing that PAM did not induce inflammation. These results open a new research field by using PAM to the development novel treatments for COVID-19, influenza, and other respiratory diseases.

## Introduction

In physics, plasma is defined as the fourth state of matter where a significant part of a gas contains atoms, radicals, ions and molecules in ground and excited states, with an equilibrium between densities of negative and positive particles^1^. Examples of plasmas are found in nature as lightnings, northern lights, stars and also in a wide range of technological applications from nuclear fusion to illumination. Such a broad spectrum of plasmas responds to the distribution of energy among species of molecules and particles that compose them. In the case that concerns us here, the plasmas of interest are those called Cold Atmospheric Plasmas (CAP). These are strongly out of thermodynamic equilibrium and they can transmit a relatively small amount of thermal energy to other bodies or materials. Indeed, under these circumstances it is not possible to use the concept of temperature strictly. The abundance of Reactive Oxygen and Nitrogen Species (RONS) in these plasmas and its low heat transmission do CAPs excellent for use in biological, medical, veterinary, food, and agronomic applications^2–7^. There is a wide international consensus about the effectiveness of RONS delivered by CAPs to deactivate bacteria, fungi, cancer cells and viruses^8–14^. The active principle is oxidative stress that RONS produce on microorganisms in a synergistic action between Oxygen and Nitrogen radical compounds^15–19^. As is well known, oxidative stress plays a key role in cell redox biochemistry. In fact, cells have a higher threshold of oxidative stress tolerance, respect to bacteria, fungi, and viruses due to structural reasons and stimulation of its self-repair mechanisms^20–22^. The antiviral capacity of CAPs against SARS-CoV-2 has been recently investigated showing an interesting potential^23–25^.

On the other hand, there is an alternative way to deliver RONS from a plasma on a surface by using liquids (for example, water or saline solutions) that have been exposed to a CAP long enough to absorb a certain RONS concentration. These liquids are given the generic name of Plasma Activated Media (PAM) and are gaining an increasing interest in medicine where for example, in-vitro and in-vivo results on cancer cells are relevant^13,14,26–29^. Although CAPs and PAMs share the same active principle of oxidative stress, PAMs have an interesting complementary advantage respect to CAPs: its capability to reach body cavities that are inaccessible for CAPs applicators systems. The possibility ot use PAMs against SARS-Cov-2 has only been recently proposed by experiments with virus and isolated pseudo-virus^30,31^ and its capability to be applied on living cells and complex body cavities had inspired our work. In this paper we show in-vitro experiments demonstrating the vulnerability of SARS-CoV-2 and PR8 H1N1 influenza A virus to PAMs with a minimal or neglectable damage to healthy cells with especial emphasis on non-inflammatory processes. Experiments with a fine aerosol of PAM on virus samples strongly aim to possibilities of developing a wide spectrum treatment on the base of a simple nebulization technique for respiratory tract diseases as COVID-19, flu, and others.

## Results

To study the effect of RONS present in the activated water on the infectivity of SARS-CoV-2 we performed a series of experiments comparing virus titers before and after contact with the activated media. Two types of PAMs were used for the experiments: Plasma Activated Water (PAW) made with distilled water and buffered PAW (bPAW) made by buffering PAW with 10% of 10X PBS in volume. The pH and RONS concentrations of used PAMs are shown in Table 1.

**Table 1:** PAW is deionized water activated by cold atmospheric plasma and bPAW is a solution of 90% of PAW buffered with 10% of PBS10X in volume.

PAM	pH	$H_{2}O_{2}$ (mg/l)	$NO_{3}^{-}$ (mg/l)	$NO_{2}^{-}$ (mg/l)
PAW	2.1	176	> 500	< 0.5
bPAW	5.2	174	487	< 0.5

**Figure 1:**
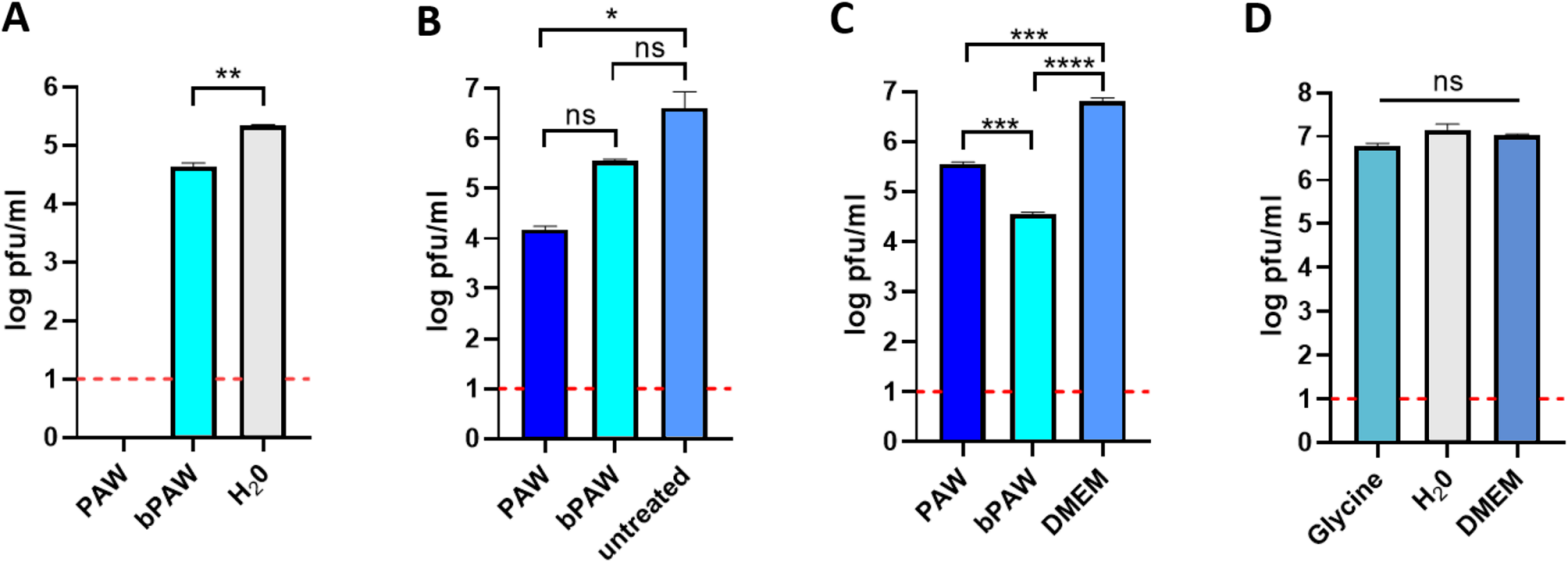
Effect of plasma activated water on SARS-CoV-2 infectivity. The figure depicts representative titration experiments of SARS-CoV-2 inocula subjected to different treatment conditions. (**A**) Mean ± SD viral titers upon incubation with plasma activated water (PAW) or phosphate buffered (bPAW) for 1 h at room temperature. The treated samples were frozen and thawed once before subjected to viral plaque assay on Vero E6 cell monolayers. Distilled water (H20) was used as a non-plasma-activated medium. **: p<0.01 unpaired (two-tailed) t-test (t = 15.22; df = 2). (**B**) Mean ± SD viral titers upon incubation with plasma activated water (PAW) or phosphate buffered (bPAW) for 30 min at room temperature and directly subjected to plaque assay without previous freezing. One-way anova (F = 14.20; p value 0.0295) with Tukey’s post-hoc test. *p<0.05; ns: non-significant. (**C**) Mean ± SD viral titers upon virus adsorption to cells and two consecutive washes with each indicated medium. Dulbecco’s modified Eagle medium (DMEM) was used as washing control. One-way anova (F = 939.4; p value <0.0001) with Tukey’s post-hoc test. ***p<0.001; ****p<0.0001. D. Mean ± SD viral titers upon incubation with media with different pH. One-way anova (F = 8.649; p value 0.0568) with Tukey’s post-hoc test. Ns: non significant. Dotted red line depicts the limit of detection of the plaque assay. Generated with INKSCAPE 1.1 (www.inkscape.org).

**Figure 2:**
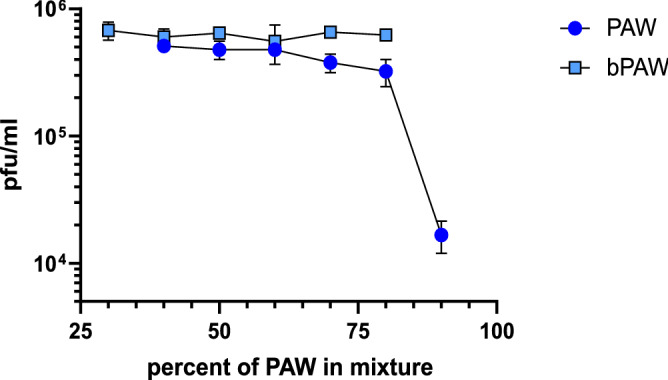
Effect of PAW concentration on SARS-CoV-2 infectivity. A fixed amount of virus was incubated for 30 min with dilutions of PAW in distilled water or in PBS. The virus mixtures were then titrated by plaque assay. The data was used for non-linear curve fitting (inset) to estimate the concentration of PAW that reduces by half the virus titer. Percentages refer to the final concentration of PAW in the mixtures that were titrated in cell cultures. Generated with INKSCAPE 1.1 (www.inkscape.org).

**Figure 3:**
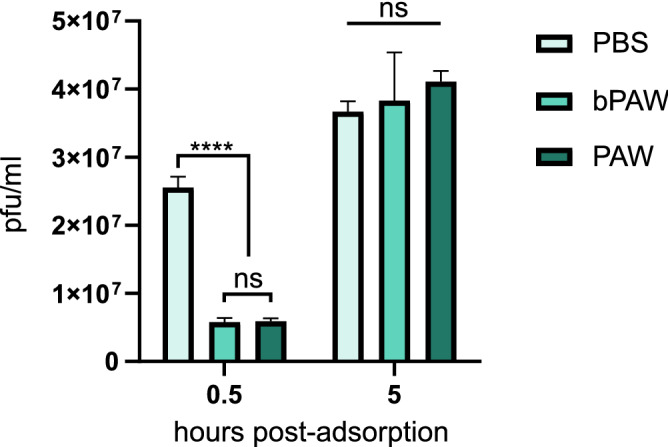
Effect of PAW ultrasonic nebulization on SARS-CoV-2 infectivity. The figure depicts the results for a representative experiment. Serial dilutions of SARS-CoV-2 inoculum were adsorbed to Vero E6 cell monolayers for either 0.5 or 5 h before nebulization with different media. Nebulization was maintained for 10 min before washing with DMEM. Viral titers were then estimated by plaque assay. PBS nebulization was used as a negative effect control. Mean ± SD viral titers are shown. Two-way anova with Tukey’s post-hoc test. ****p<0.0001.; ns: non-significant. Generated with INKSCAPE 1.1 (www.inkscape.org).

**Figure 4:**
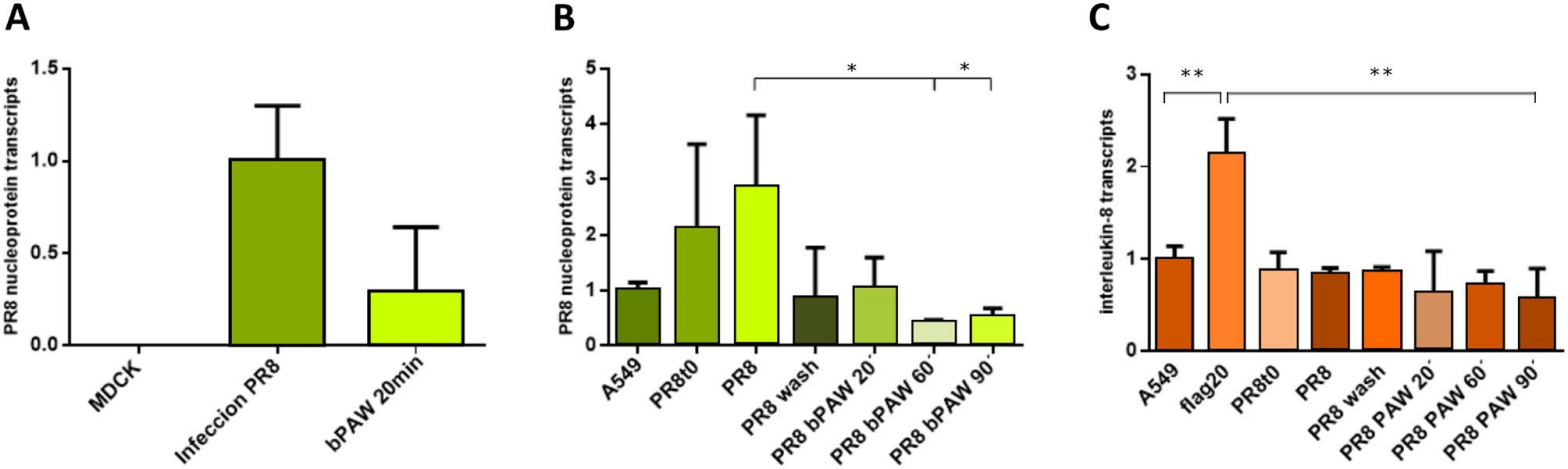
Effect of plasma activated water on infectivity and inflammation caused by influenza PR8 H1N1 virus. (**A**) Results of inhibition of PR8 H1N1 virus replication in the canine kidney MDCK cell line obtained after infection with PR8 and subsequent treatment with bPAW. The figure depicted the PR8 nucleoprotein transcript level (arbitrary units, a. u.). (**B**) bPAW Inhibition of the internalization of the PR8 virus measured by nucleoprotein RNA transcripts (a. u.). A549 human lung cells were subjected to PR8 H1N1 infection for 1 h and subsequently treated with bPAW for 20, 60 and 90 min. (**C**) Study of inflammation produced by bPAW treatment in the A549 human lung cell line. The panel denoted the normalized level of interleukin 8 RNA transcripts. All figures represent normalized values (Mean ± SD). Statistical analysis was carried out using one-way anova with Tukey’s post-hoc test, *p<0.01; **p<0.001. Generated with INKSCAPE 1.1 (www.inkscape.org).

Figure 1 shows the results of titration experiments of SARS-CoV-2 inocula subjected to different treatment conditions. SARS-CoV-2 virus was diluted to 10% in either distilled water, PAW or bPAW. The mixtures were incubated for 1 h at room temperature with constant rocking. In this experiment the samples were frozen until use. After thawing, decimal dilutions were added to fresh monolayers of Vero E6 cells and adsorbed for 1 h. The viral titers were estimated by plaque lysis assay. The treatment with PAW completely abolished the infectivity of SARS-CoV-2 while incubation with buffered PAW induced a reduction of 80% in the viral titers, when compared with the titers obtained after incubation with distilled water (Fig. 1A). The complete abolition of infectivity (no plaques were visible at the lowest dilution) upon PAW treatment could be due to the overextended contact period considering the time to completion of freezing and thawing. For a more controlled estimation of the infectivity loss, the treatment was performed for only 30 min immediately prior to incubation with Vero E6 cells. In this experimental setting we observed a titer reduction of around 90% and 99% upon treatment with bPAW or PAW, respectively (Fig. 1B). This result clearly demonstrated the deleterious effect of PAW on the virus viability and that this effect was slightly ameliorated after phosphate buffering of the medium. To test if both PAMs could have an effect right after virus-cell attachment, Vero E6 cells were incubated with serial SARS-CoV-2 virus dilutions for 1 h. Infected cells were then subjected to two consecutive washes with either PAW or bPAW and the titers estimated 48 h later as before. The reductions in titers were of 99.5% and 95% for bPAW and PAW respectively, indicating that PAMs efficiently altered the SARS-CoV-2-cell interaction (Fig. 1C). As aforementioned, the active principle of PAW is due to concentration of RONS, but low pH could play a significant role in its antiviral effect. Since most viral glycoproteins rely in a pH-dependent conformational change for viral and endosomal membrane fusion upon internalization we wondered if the extremely low pH of PAW could be responsible for the observed infectivity loss upon treatment. To estimate the influence of pH in the attachment efficacy a similar virus dilution experiment was performed, using different media with low to physiologic pH. The effect of lower than physiologic pH (5.5 or 2.7) resulted in a 22% and 58% infectivity reduction, respectively (Fig. 1D). These data point out to a combined effect of RONS and low pH as drivers for the loss of infectivity of SARS-CoV-2 particles and might explain the differences observed upon PAW and bPAW treatment (pH 2.1 and 5.2, respectively). Taken together, these data demonstrate the negative impact of plasma activated media on the viability of SARS-CoV-2 particles in cell culture. To make an estimation of the concentration at which PAW exerts half of its maximal SARS-CoV-2 infectivity inhibition ($\hbox {IC}_{{50}}$) a fixed amount of virus inoculum was incubated with decreasing concentrations of PAW, ranging from 90% to 30% in distilled water or PBS (Fig. 2). Upon non-linear curve fitting, the estimated $\hbox {IC}_{{50}}$ of PAW was 83.72%. Since the maximal concentration of PAW resulting after buffering with phosphate salts and incubation with the virus inoculum was below this threshold (80%) it could not be observed a clear dilution effect using this treatment, precluding the generation of a proper curve fitting for $\hbox {IC}_{{50}}$ estimation. The effect of different PAW concentrations on cultured Vero E6 cells was also assessed. The incubation of cells for 10 min with PAW dilutions resulted in full viability loss due to the non-isotonic nature of the media (not shown). One potential application of PAW would be to reduce the viral load in infected mucosal tissues. For human respiratory viruses such approach could rely on aerosol treatment. Thus, nebulization experiments with PAW were carried out with samples of Vero E6 cells infected with serially diluted SARS-CoV-2 inoculum. Vero E6 cell monolayers were used, as in previous experiments. The inoculum was allowed to contact cells for 0.5 h or 5 h, representing either preinternalized or internalized virus particles, respectively. After exposure to ultrasonic nebulization for 10 minutes, the treated cells were processed as for plaque lysis assay. Figure 3 shows that when the virus remained cell-attached the nebulization with both PAW and bPAW was able to alter the infectivity of inoculum, allowing for a reduction of about 77% with respect to infected cells nebulized with PBS. This effect was not evident after virus internalization suggesting that the SARS-CoV-2 inhibitory effect of PAW is more related to host cell-virus alterations rather than affecting other downstream processes in the viral cycle.

On the other hand, infection tests with the PR8 H1N1 virus, responsible of common Influenza or flu, were performed by using the Madin-Darby canine kidney (MDCK) cell line. Once infected with the PR8 H1N1 virus at multiplicity of infection (MOI) of 5, the cells were incubated for 24 h in a regime of free virus multiplication and subsequently exposed to bPAW treatment for 20 min. Figure 4A shows the results of inhibition on replication by measuring the level of PR8 virus nucleoprotein (N) messenger RNA transcripts by quantitative PCR (qPCR). Columns represent the reference sample (MDCK), cells infected with PR8 (Infection PR8) and infected cells that were treated with bPAW for 20 min of exposure (bPAW 20 min). The antiviral activity of PAW against PR8 H1N1 is observed because the level of nucleoprotein (N) transcripts decreases by 71% with respect to the untreated sample. Figure 4B shows results of the internalization inhibition for PR8 virus in the A549 human cell line subjected to infection for 1 h and subsequently treated with bPAW during 20, 60 and 90 min. The amount of virus nucleoprotein messenger RNA transcripts was measured by quantitative PCR (qPCR). As it can observed, two consecutive washes with bPAW reduced significantly the internalization of PR8. A similar result was observed when cells were incubated for 20 min. However, cells incubated with bPAW for 60 min led to the complete elimination of infectivity on A549. In all cases in which bPAW was used, virus internalization was inhibited

Additionally, a study of inflammation due to PAM treatment was carried out with the human A549 lung cell line. In this case, the amount of interleukin 8 messenger RNA transcripts was measured by qPCR, a precursor cytokine of inflammation that makes it possible to establish whether inflammatory processes occurs^32^ . Figure 4C shows the values obtained in A549 cells submitted to the same treatment of that described for Fig. 4B. Results showed that the inflammatory action of PAM on A549 lung cells was not appreciable after 90 min of treatment whereas flagellin was able to increase the interleukin 8 transcript level, being statistically significant when compared to any other sample. Therefore, it is demonstrated that bPAW does not produce inflammation.

**Figure 5:**
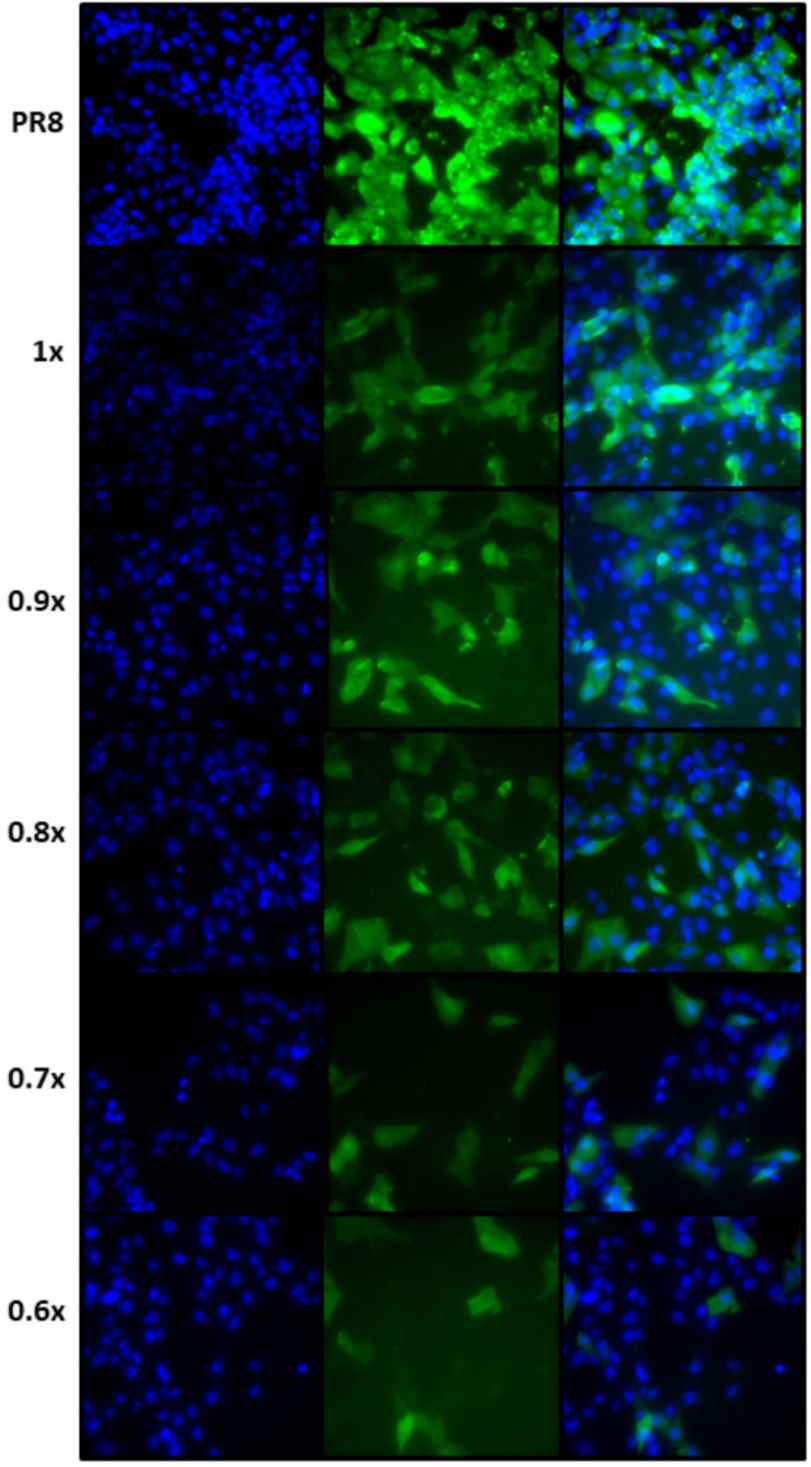
Immunofluorescence of MDCK infected with PR8 (MOI of 0.25) after the treatment with different PAW-PBS dilutions. The images are representative from three experiments. Results showed a reduction of infection between PR8 infected cells and PR8 infected cells and further treated with PAW-PBS. Generated with INKSCAPE 1.1 (www.inkscape.org).

**Figure 6:**
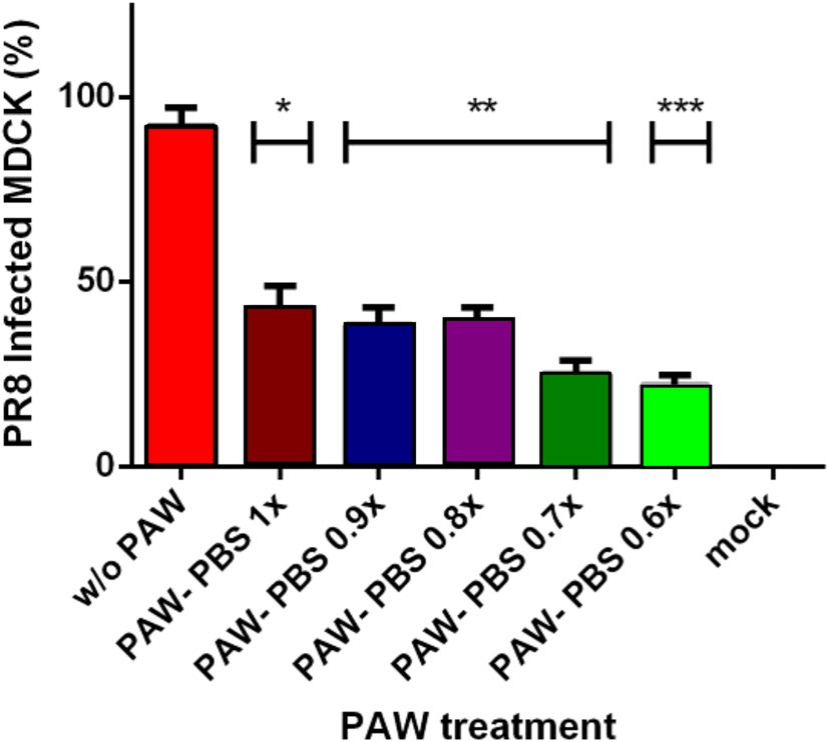
Counting of infected cells with PR8 without and with different PAW-PBS dilutions. Cells were infected with a MOI of 0.25 for 1 h, then submitted to the different treatments, and led the progression of the infection for 24 h. Immunofluorescence was carried out as described in methods and final counting of infected cells versus non-infected cells. Infection was reduced by more than a half (53%) with the lowest dilution of PAW-PBS used (90%PAW-0.1x PBS). Statistical analysis was carried out using unpaired t test (*p<0.05, **p<0.009, ***p<0.0004). Generated with INSKAPE 1.1 (www.inkscape.org).

**Figure 7:**
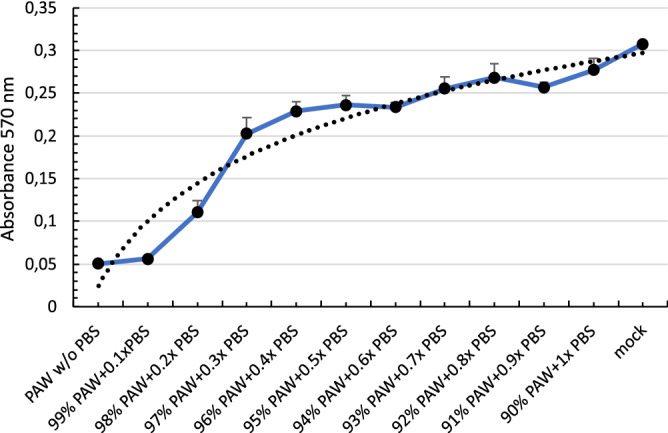
Effect of PAW concentration on MDCK viability. Cells were incubated for 20 min with dilutions of PAW-PBS, ranging from 100% to 90% of pure PAW. Cell viability was determined by MMT assay. Absorbance values were used to calculate an exponential curve fitting and to estimate the concentration of PAW-PBS that causes 50% of citotoxicity (CC50). Generated with INKSCAPE 1.1 (www.inkscape.org).

**Figure 8:**
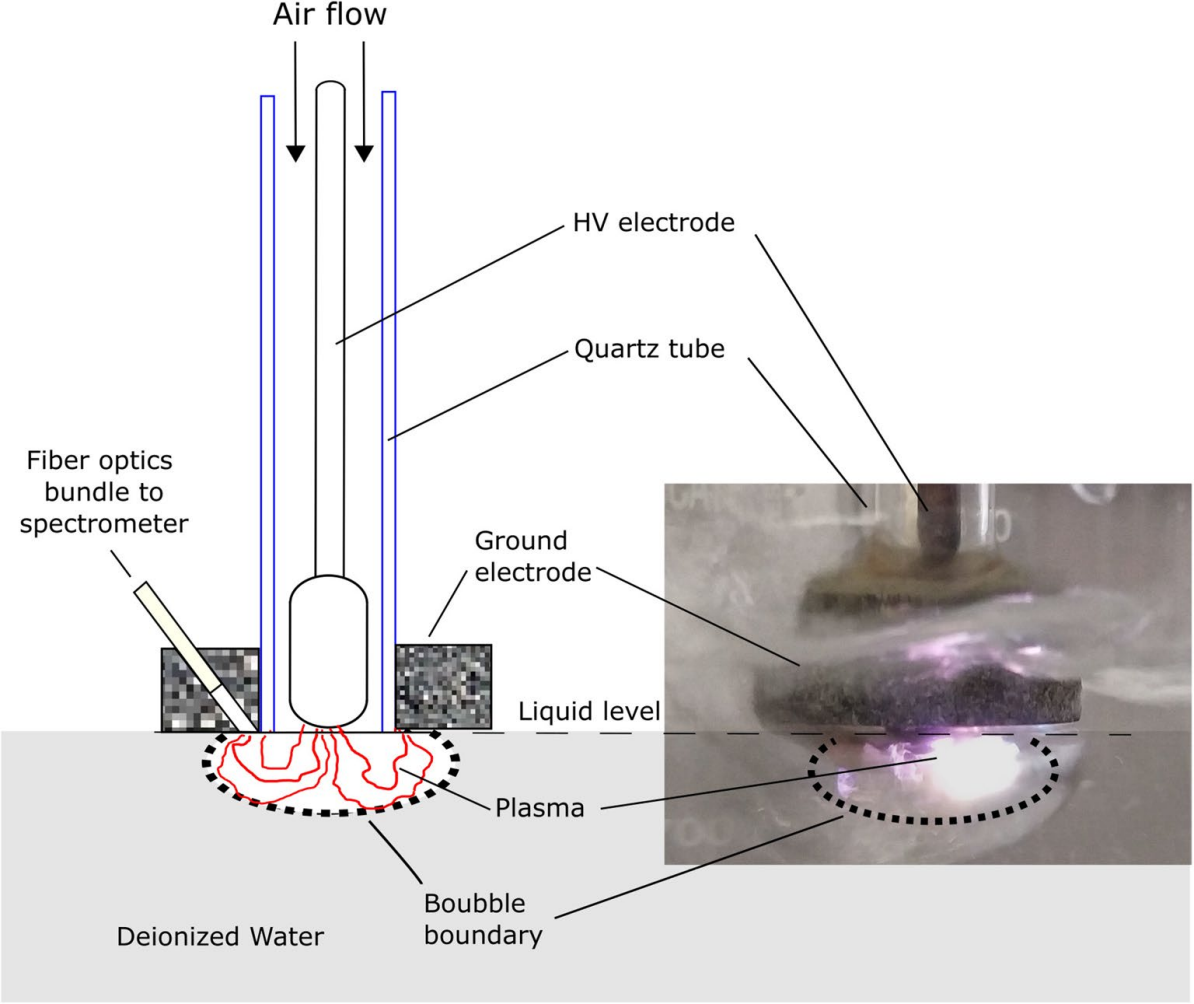
Experimental setup for PAW production. A cold out of equilibrium plasma is produced inside of bubbles of air. Generated with INKSCAPE 1.1 (www.inkscape.org).

**Figure 9:**
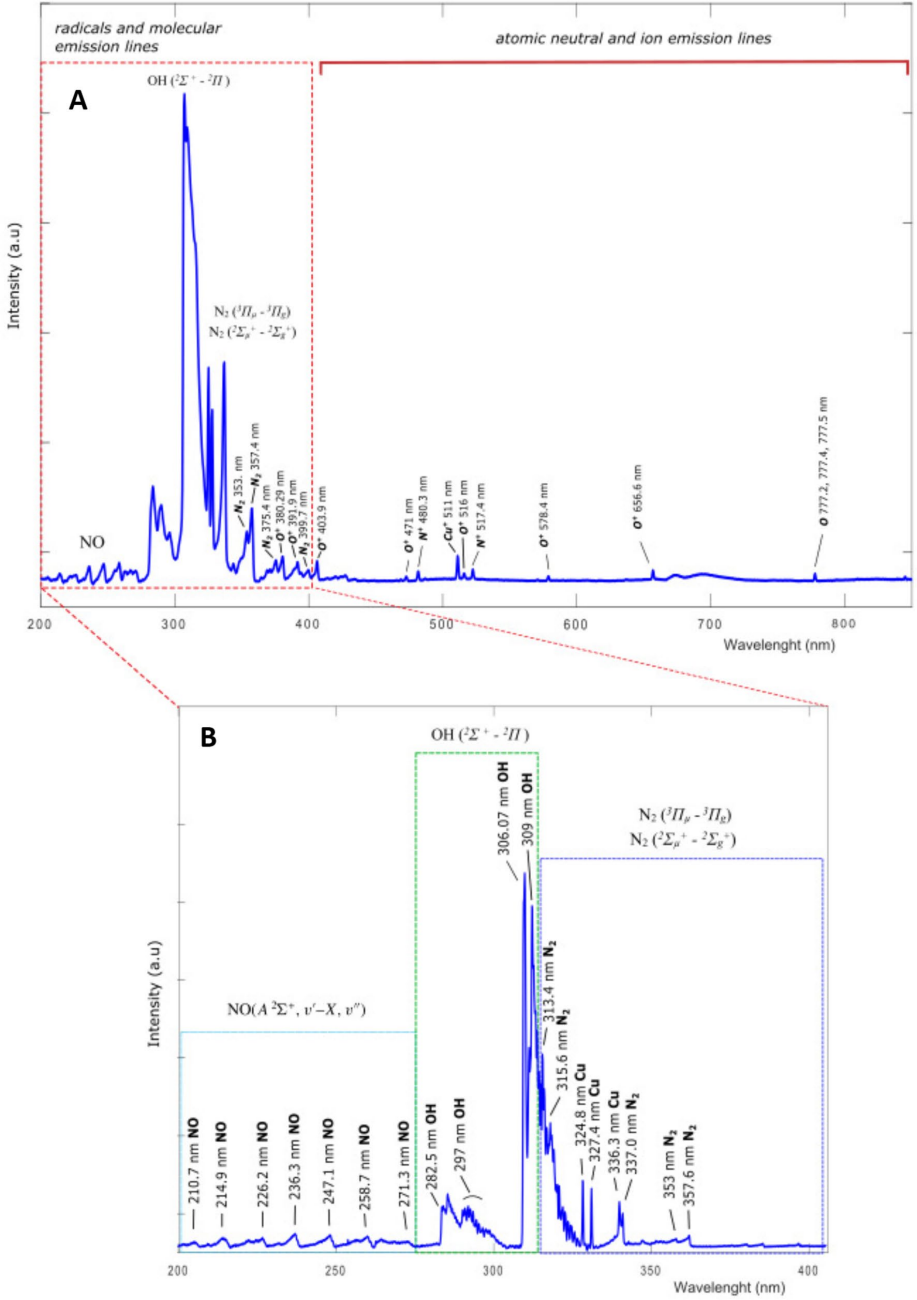
Emission spectra obtained from the jet of cold atmospheric plasma while deionized water is activated: (**A**) UV-VIS 200–800 nm range and (**B**) UV 200–400 nm range. The groups of lines corresponding to molecular and atomic transitions are shown in boxes indicating their origin. Generated with INKSCAPE 1.1 (www.inkscape.org).

In addition to qPCR viral load of PR8, the production of infective PR8 was also determined. For that, MDCK cells infected with PR8 were treated with PAW at different dilutions, and the infection after 24h was observed by using indirect immunofluorescence. A representative image of the results is depicted in Fig. 5. Global results showed a 92.04% of infection in PAW-untreated MDCK cells infected with PR8 while those cells treated with PAW-PBS 1x (bPAW) reduced the infection close to 43%, being a 53% of infectivity reduction (Fig. 6). When PAW solution is more concentrated due to lower PBS concentration, PAW gained effectiveness reducing PR8 infection from 92.04% (PR8 w/o PAW) to 22% (94% PAW-PBS 0.6x), which led to 76% less infection than PR8. MDCK cells showed distinct viability after the treatment with different PAW media with an exponential curve (Fig. 7), ranging from pure PAW (very low cell viability) to PAW-PBS 1x (bPAW) showing a viability close to that of mock cells. The dilution bPAW represented a dilution 1/10 of pure PAW. Curve fitting to an exponential equation yield a R2 = 0,9243. As result, CC50 estimation value is obtained between the dilutions 98% PAW+0.2x PBS and 97% PAW+0.3x PBS. The efficacy of PAW is gained with higher percentage purity, however, this also leads to higher cytotoxicity. Therefore, the highest dilution of PAW (bPAW) had the lowest cytotoxicity, and would reach the $\hbox {EC}_5$ $_0$.

## Discussion

In this work the vulnerability of SARS-CoV-2 and PR8 H1N1 virus to Cold Atmospheric Plasma Activated Media (PAM) is demonstrated with in-vitro experiments for isolated virus and infected cells. Our experiments showed a limited influence of pH over the full antiviral effect and a non-inflammatory action on cells by PAM. To explore the possibilities of developing antiviral treatments for respiratory diseases, experiments with PAM aerosol or spray were conducted with encouraging results. One limitation of our study is that we did not identify which of the reactive species generated in PAW are more determinants for the inactivation effect. It is reasonable to speculate, however, that the hydrogen peroxide concentration of our PAMs (around 0.02% w/w) is probably too low to be responsible for the titer drop of the virus. In this sense it was recently shown that a 0.5% w/w solution was not enough to significantly reduce SARS-CoV-2 titers, and only higher concentrations (3% w/w) could show clear titer drops upon 5 minute contacts^33^. In these experiments higher titer drops were shown when lowering the pH of the solution. These data point out to a synergistic effect of $\hbox {H}_2$ $\hbox {O}_2$, low pH and RONS determining the virucidal properties of the PAMs tested in this work.

During the editorial submission of this manuscript Szili et al.^30^ reported the inhibition of SARS-CoV-2 infectivity with two acetyl donors (TAED and pentaacetate glucose) present in an aqueous solution that was activated with cold plasma (PA-TP). PA-TP medium contains peracetic acid in addition to RONS or $\hbox {H}_2$ $\hbox {O}_2$ and was able to reduce the virus loads in higher percentages with respect to a distilled water activated control (PA-DIW). Altogether these data confirm the potential of PAMs as appealing viral inactivation alternatives to other less environmental friendly decontaminating reagents.

On the other hand, while the results limiting coronavirus or influenza virus replication were clear, cold atmospheric plasma has not always an antiviral effect. There are some studies demonstrating that using CAP increases the infectious bovine rhinotracheitis virus (IBRV) in MDBK cells. There is a synergic mechanism shared by IBDV and CAP20. As result, IBDV infected and CAP treated cells were in a G2/M arrest stage. Moreover, CAP suppressed Interferon Regulatory Factor 7 (IRF7) in those cells infected by IBRV leading to an inefficient antiviral state. On the other hand, human adenovirus susceptibility to CAP is serotype-dependent^34^. So, CAP had an antiviral effect on HAdV-5 and HAdV-37, a neutral effect on HAdV-4 and HAdV-50, and a proviral effect on HAdV-20 and HAdV-35 serotypes. Therefore, these studies suggest that it is necessary to determine the antiviral capability of CAP for each virus family. In contrast, the advantages of PAMs aim to its use as a possible contribution in the fight against COVID-19 and influenza among other respiratory diseases. Oxidative stress (OS) is part of one natural cell’s biochemistry, and could play a key role to develop treatments for contention of respiratory viruses. Moreover, the possibility to apply a PAM in an aerosol mist to the upper respiratory tract with a nebulizer device that is universally available is extremely attractive due its simplicity. Considering that main multiplication and propagation of SARS-CoV-2 takes place during the first weeks of COVID-19 symptoms in the upper respiratory tract^35^, the application of a mist of PAM could help to contain the virus propagation while patients develop a better immune response. PAM would be effective against all SARS-CoV-2 variants of concern that could arise and escape neutralizing antibodies, and against seasonal and highly pathogenic influenza viruses as well. Moreover, it is also important to highlight the broad-spectrum action of PAM on bacteria and fungi. Especially considering the patients who suffer complications with these infections simultaneously with COVID-19, the use of PAMs could make a great benefice.

We cannot forget that SARS-CoV-2 is only one of viruses in a chain of outbreaks that is affecting us in last decades and everything aims to that more viruses will came soon^36^. The development of wide range treatments to content virus propagation while new vaccines are developed has a high priority. In this way, the use of aerosol of Plasma Activated Media could represent an important contribution.

## Methods

### Facilities

The experiments were conducted in the biological security facility of Animal Health Research Center (CISA, INIA-CSIC) and the Department of Biotechnology, at National Research Institute of Agricultural and Food Technology (INIA-CSIC) in Madrid, Spain, where SARS-CoV-2 strain/NL/2020 and influenza A/PR/8/34 (H1N1) are available for R&D purposes^37^. PAMs were produced in the laboratories of ION BIOTEC S.L. in Puertollano, Spain (www.ionbiotec.com).

### PAW and bPAW

Plasma Activated Water (PAW) was obtained by direct exposure of deionized water (conductivity $\le$ 10 $\mu$S/cm) to a cold atmospheric air plasma discharge as shown in Fig. 8. Coaxial electrodes separated by a quartz tube of 1 cm of inner diameter and 1 mm wall thickness are placed in contact with the liquid surface. The electrodes are energized by 20 kV power supply while air flow is 5 l/min. While plasma is interacting with the water surface, a fiber optics bundle allows to obtain UV-VIS and UV spectra plasma-water interface. We used a UV-VIS and VIS spectrometers from BWTEK Inc. Models Exemplar-LS BRC115P-U-UV (200–400 nm) and BRC115P-U-UV/VIS (200–800 nm) respectively with an Ocean Insight model QP1000-2-SR (200–900 nm) fiber optics. The exposure time between plasma and water is of 30 min to obtain 200 ml of PAW with the concentration of RONS detailed in Table 1. Concentrations of hydrogen peroxide, nitrate, nitrite and pH were measured by using color fixed indicator strips Quatofix by Macherey-Nagel: Peroxide 1000, Nitrate-Nitrite ans pH-Fix 0-14 respectively. The readings of color changes in the strips were performed by using a reflectometer with auto-calibrated microprocessor control Quantofix Relax. Buffered Plasma Activated Water (bPAW) was made by buffering PAW with a 10% in volume of 10XPBS. Figure 9A shows a wide range VIS-UV spectrum between 200–800 nm of wavelengths where radical, molecular, and atomic lines are observed. Figure 9B shows a narrower range spectrum from UV between wavelength of 200 and 400 nm obtained with other spectrometer with higher resolution to observe a more detailed molecular and radical emission from RONS like NO, OH and $\hbox {N}_{{2}}$ lines. The activation rate of PAMs strongly depends on the relative abundance of RONS in the plasma-water interface as is shown in the spectra. On-fly measurement of RONS lines intensities while activation is produced allows to optimized plasma generation parameters as air flow, voltage, frequency etc. to obtain a higher transference of RONS to the liquid. In our case, typical activation rate values of 1.2 mg/min of hydrogen peroxide and 4.0 mg/min of nitride are reached. It is worthy to mention that these water activation rates are two orders of magnitude higher than typical of other devices^38^.

### Cells and virus

Vero E6 cells (ATCC/CRL/1586) were routinely maintained in Dulbecco’s modified Eagle’s medium (DMEM) containing supplements (10% foetal bovine serum, 2 mM glutamine, 100 U/mL penicillin, 100 mg/mL streptomycin). Cells were routinely incubated at 37$^\circ$C in a CO2 atmosphere with 95% humidity. The SARS-related coronavirus 2 strain NL/2020/ (BetaCoV/Netherlands/01) was used. A virus stock was generated by infecting Vero E6 cells at low infection multiplicity (0.01 PFU/cell). When cytopathic effect (cpe) was evident (48–72 h post infection) the supernatants were clarified by low-speed centrifugation and stored in aliquots at -80$^\circ$C until use.

**Table 2:** Primers sequences used in this study for quantitative PCR.

Name	Forward	Reverse
GAPDH human	5’CAAGGATATCCGTCGTGGCA	5’ACAGCGAAACGACCAAGAGG
GAPDH canine	5’CCTGGAGCATCATGGCGTG	5’GCTGGAGAGTGCTGTGGAAGAACATATAG
Nucleoprotein PR8 H1N1	5’GGGAGAAAATGATCAAGATGCTCGT	5’GCAGACATGCCTCCTTGTTGG
Interleukin 8	5’AGAGACACTGAGATCATTGCCAC	5’AGAGACACTGAGATCATTGCCAC

### MDCK cell viability after treatment with PAW

Measurements of cell viability were performed using the CyQUANTTM MTT Cell Proliferation Assay Kit (Invitrogen, Cat. V13154) by quadruplicate. The assay involves conversion of water soluble MTT (3-(4,5-dimethylthiazol-2-yl)-2,5-diphenyltetrazolium bromide) to an insoluble formazan. PAW dilutions were made with 10X PBS as following: (1) pure PAW, (2) 99% PAW+0.1x PBS, (3) 98% PAW+0.2x PBS, (4) 97% PAW+0.3x PBS; (5) 96% PAW+0.4x PBS; (6) 95% PAW+0.5x PBS; (7) 94% PAW+0.6x PBS; (8) 93% PAW+0.7x PBS; (9) 92% PAW+0.8x PBS; (10) 91% PAW+0.9x PBS; and (11) 90% PAW+1x PBS; which is termed bPAW. MDCK cells were seeded ($10\times 10^3$/well) in p96 plate the day before test. Next day, cells were washed with PBS and then incubated for 20 min with 100 $\mu$l of each PAW mixture. After that, the protocol was carried out following the manufacturer’s instructions. For CC50 estimation we followed a four-parameter logistic regression model. The calculations were performed using an online tool ”Quest Graph$^{\mathrm{TM}}$ CC50 Calculator “AAT Bioquest, (https://www.aatbio.com/tools/ic50-calculator). Values were normalized dividing by the largest response.

### SARS-CoV-2 plaque assay

The titer of the SARS-CoV-2 stock was determined in Vero E6 cells by conventional plaque assay. Samples were subjected to 10-fold serial dilutions and added to each well. After adsorption of the virus inoculum at 37$^\circ$C, the cells were washed with medium and a semisolid mixture of 1% Carboxy-methyl cellulose (CMC) in serum supplemented DMEM added to each well. 72 h later the wells were examined for the presence of virus induced lysis plaques. The cells were fixed with 10% formaldehyde solution, the semisolid medium removed, and the fixed cultures further stained with 2% crystal violet solution. After washing out the staining solution, plaques were visually inspected and counted in those wells displaying more than 30 lysis plaques. The viral titers were estimated by the following formula: (number of plaques x sample dilution factor)/sample volume (in mL). All SARS-CoV-2 culture procedures were conducted in an enhanced biosafety level 3 laboratory (BSL3+). All personnel wore powered air-purifying respirators (3M) incorporated into Prochem suits. Manipulation of live infectious virus was performed inside a biosafety class-II cabinet.

### Titration of media-treated SARS-CoV-2

For most experiments, the SARS-CoV-2 stock was diluted 1/10 in each different media and incubated at room temperature with constant shaking under a biosafety class-II cabinet. Incubation times ranged from 3 to 60 min. Upon incubation, the samples were either ten-fold diluted in DMEM and applied directly to Vero E6 cultures or immediately frozen for further use. Titration of residua virus upon treatment was performed by plaque assay as above.

### Influenza infection determined by indirect immunofluorescence

MDCK cells ($50\times 10^3$ /well) were plated in 24-well plates in DMEM containing 5% FBS (Gibco) supplemented with L-glutamine (2 mM, Gibco), and penicillin/streptomycin (Pen/Strep, 100 U/ml). The following day, cells were infected with the influenza strain A/PR/8/34 at MOI of 0.25 for 1 h at 37$^\circ$C. Then, cells were further submitted to different treatments for 20 min by using 500 $\mu$L of pure PAW and different dilutions with 10X PBS: (1) pure PAW, (2) 99% PAW+0.1x PBS, (3) 98% PAW+0.2x PBS, (4) 97% PAW+0.3x PBS; (5) 96% PAW+0.4x PBS; (6) 95% PAW+0.5x PBS; (7) 94% PAW+0.6x PBS; (8) 93% PAW+0.7x PBS; (9) 92% PAW+0.8x PBS; (10) 91% PAW+0.9x PBS; (11) 90% PAW+1x PBS; (12) mock. After PAW treatment, cells were incubated for 24 h at 37 $^\circ$C with DMEM supplemented with 2% foetal bovine serum, 2 mM glutamine, 100 U/mL penicillin, 100 mg/mL streptomycin. Then, media was removed, the cells washed with PBS, and fixed with paraformaldehyde 4% for 10 min. MDCK cells were washed with PBS and then treated with ice-cold methanol for 10 min. After that, MDCK were washed again with PBS and then blocked for 60 min with PBS-tween20 (0.1%)-triton X-100 (0.3%). Cells were incubated with anti-PR8 antibody at 1/300 for 60 min (a gift of Dr. Aitor Nogales, CISA, INIA-CSIC). After washing with PBS, cells were incubated with a goat anti-mouse A-488 (Invitrogen, $\#$ A28175) at 1/1000 for 30 min in dark. For nuclei staining, cells were incubated with Hoechst at 1/1000 for 10 min and further washed. Cells were visualized by fluorescence microscopy using a Nikon Eclipse TE2000-E microscope. For each treatment, the percentage of infection was calculated by using (infected/total cells) $\times$ 100. Cells were counted using ImageJ software (https://imagej.nih.gov/ij/) and the plugin cell counter taking into account three different fields for each treatment. These data was used to estimate $\hbox {IC}_{{50}}$. The calculations were performed using described above (https://www.aatbio.com/tools/ic50-calculator). Values were normalized dividing by the largest response.

### PR8 infection and induced-inflammation determined by transcriptomic profile on MDCK and A549

Total RNA was extracted using the FavorPrepTM Tissue Total RNA Mini Kit (Favorgen) according to the manufacturing specifications, performing on-column deoxyribonuclease I (Applied Biological Materials Inc.) treatment to remove genomic DNA that might interfere with the PCR reactions. RNA was quantified with the NanoDropTM. One Spectrophotometer (Thermofisher) and cDNA synthesized using the HighCapacity cDNA reverse Transcription Kit (Applied Biosystems) and oligo (dT) primer. The mRNA levels of different immune genes were determined by quantitative PCR (qPCR) with a 7500 Fast Real-Time PCR System instrument (Applied Biosystems) using 2x qPCRBIO SyGreen Mix Lo-ROX (PCR Biosystems). The primers were used at a final concentration of 400 nM (see Table 2). The running conditions were as follow: 30 s at 95 $^\circ$C, followed by 45 cycles of 3 s at 95 $^\circ$C and 30 s at 60 $^\circ$C. A melting curve analysis was included for each run to ensure the specificity of the reaction. Gene expression was corrected by the housekeeping GAPDH gene. Fold change ($2^{-\Delta \Delta \mathrm{Ct}}$) of transcript level for each gene was calculated by subtracting the relevant mean $\Delta$ $\Delta$Ct values obtained in their respective control group. The reported changes in transcript levels are from median values calculated for the whole group that comprised 4 replicates.

### Nebulization chamber

The Nebulization chamber was made by ION BIOTEC S.L. The chamber is a 12 liters transparent plastic box with an airtight lid, and it allows to observe while the aerosol is introduced from a hose connected to a standard ultrasonic nebulizer for aerosol therapy LAICA Advance Technology Inhalation Series MD6026P. A holder 9 $\times$ 13 cm for 12 wells with the samples to be treated can be accommodated inside. The nebulizer is loaded with the PAM to be projected and it can produce a fine aerosol of droplets of up to 5 microns in air with a flow of 0.5 $cm^{3}/min$. The aerosol travels through hose to the chamber where it is deposited on the surface of the samples. The chamber is equipped with four fine particle filters attached to walls that maintain the atmospheric pressure inside but prevent the mist loss to outside. The mean density value of the aerosol inside the chamber is estimated in 1 $mg/cm^{3}$.

### Nebulization experiments, Vero E6 cell viability and $\hbox {IC}_\mathbf{50 }$ estimation

Vero E6 cells were infected with ten-fold dilutions of SARS-CoV-2. Before nebulization, the virus was allowed to attach to cells for 30 minutes at 4$^\circ$C to avoid internalization. Another set of cells was incubated with the same dilutions and allowed to progress for 5 hours at 37$^\circ$C. Ultrasonic nebulization of media was carried out in the previously described sealed chamber for 10 min at room temperature in a safety class-II hood. Upon treatment, the cells were washed and covered with semisolid CMC medium as for plaque assay. Viral plaques were visualized after 72 h and 296 counted as described. In Vero E6 cells measurements of cell viability were performed using the CellTiter 96^®^ AQueous One Solution Cell Proliferation Assay System (Promega). PAM dilutions were made in distilled water (for PAW) or in PBS (for bPAW). SARS-CoV-2/PAM mixtures were incubated for 30 min at room temperature. Upon incubation, the mixtures were 10-fold serially diluted in DMEM 10% FBS and added to Vero E6 cell monolayers for plaque assay titration as described previously. For $\hbox {IC}_{{50}}$ estimation we used a four parameter logistic regression model. The calculations were performed using an online tool “Quest Graph$^{\mathrm{TM}}$ $\hbox {IC}_{{50}}$ Calculator, AAT Bioquest, Inc (https://www.aatbio.com/tools/ic50-calculator). Values were normalized dividing by the largest response.

### Statistical analysis

The GraphPad software (Prism 6. https://www.graphpad.com) was used for graphical representations and significance comparisons. SARS-Cov-2 data were log10 transformed before test analysis with unpaired Student’s t-test or Anova as indicated in figure captions.
